# Evaluation of the Effect of Morphological Structure on Dilatational Tracheostomy Interference Location and Complications with Ultrasonography and Fiberoptic Bronchoscopy

**DOI:** 10.3390/jcm13102788

**Published:** 2024-05-09

**Authors:** Esin Bulut, Ulku Arslan Yildiz, Melike Cengiz, Murat Yilmaz, Ali Sait Kavakli, Ayse Gulbin Arici, Nihal Ozturk, Serkan Uslu

**Affiliations:** 1 Department of Anesthesiology and Reanimation, Akdeniz University Faculty of Medicine, Antalya 07070, Turkey; esi.bulut@gmail.com (E.B.); melikecengiz@yahoo.com (M.C.); muryigit@yahoo.com (M.Y.); gulbinarici@akdeniz.edu.tr (A.G.A.); 2 Department of Anesthesiology and Reanimation, Istinye University Faculty of Medicine, Istanbul 34010, Turkey; alisaitkavakli@hotmail.com; 3 Department of Biophysics, Akdeniz University Faculty of Medicine, Antalya 07070, Turkey; nozturk@akdeniz.edu.tr (N.O.); serkanuslu@akdeniz.edu.tr (S.U.)

**Keywords:** fiberoptic bronchoscopy, complications, morphology, percutaneous dilatational tracheostomy, ultrasonography

## Abstract

**Background:** Percutaneous dilatational tracheostomy (PDT) is the most commonly performed minimally invasive intensive care unit procedure worldwide. **Methods:** This study evaluated the percentage of consistency between the entry site observed with fiberoptic bronchoscopy (FOB) and the prediction for the PDT level based on pre-procedural ultrasonography (USG) in PDT procedures performed using the forceps dilatation method. The effect of morphological features on intervention sites was also investigated. Complications that occurred during and after the procedure, as well as the duration, site, and quantity of the procedures, were recorded. **Results:** Data obtained from a total of 91 patients were analyzed. In 57 patients (62.6%), the USG-estimated tracheal puncture level was consistent with the intercartilaginous space observed by FOB, while in 34 patients (37.4%), there was a discrepancy between these two methods. According to Bland Altman, the agreement between the tracheal spaces determined by USG and FOB was close. Regression formulas for PDT procedures defining the intercartilaginous puncture level based on morphologic measurements of the patients were created. The most common complication related to PDT was cartilage fracture (17.6%), which was proven to be predicted with maximum relevance by punctured tracheal level, neck extension limitation, and procedure duration. **Conclusions:** In PDT procedures using the forceps dilatation method, the prediction of the PDT intervention level based on pre-procedural USG was considerably in accordance with the entry site observed by FOB. The intercartilaginous puncture level could be estimated based on morphological measurements.

## 1. Introduction

Percutaneous dilatational tracheostomy (PDT) is a bedside procedure performed in the intensive care unit (ICU) to establish airway patency and ventilate the patient. The requirement for prolonged mechanical ventilation is the most common indication for PDT in critically ill patients. Other indications include relieving upper airway obstruction, preventing laryngeal and upper airway damage due to prolonged translaryngeal intubation, and providing easy or frequent access to the lower airway for aspiration and secretion removal [1].

PDT and the Seldinger principle were described by Ciaglia et al. in 1985 [2]. In this technique, the trachea is percutaneously perforated with a sharp, hollow trocar, through which a guidewire is advanced. The trocar is then retracted, and dilators are passed serially over the guidewire, directing the tip of the dilators into the tracheal lumen. Since then, many modifications of the Seldinger technique (variations in the method of creating and dilating the tracheal stoma) have been developed. The currently favoured PDT techniques are the multiple dilatation method (Ciaglia technique), the one-step dilatation method (Ciaglia blue rhino), the forceps dilatation method (Griggs), the phantom translaryngeal method, and the controlled rotation method (Percutwist). Although each of these techniques has advantages and disadvantages, there is no consensus on which of these methods is the best in terms of efficacy and safety. The method of choice is usually determined by subjective factors such as experience [3].

PDT has some complications that can be encountered in the early and late periods. Early complications (0–7 days of procedure) include haemorrhage, loss of airway, false mediastinal passage, hypoxia, pneumothorax, pneumomediastinum, posterior tracheal wall injury, oesophageal injury, subcutaneous emphysema, increased intracranial pressure, atelectasis, and tracheal ring fracture. During this period, the most common complication is minor bleeding, and the most serious complication is loss of the airway. Late complications (beyond day 7) include subglottic stenosis, unplanned decannulation, tracheoinnominate artery haemorrhage, tracheal cannula displacement, tracheoesophageal fistula, infection, dysphagia, persistent voice change, and delayed wound healing after decannulation [4]. The intervention site of PDT may be pivotal to avoid these complications. Relatively small-caliber upper cartilages and lower cartilages should be avoided to reduce the possibility of subglottic tracheal stenosis and to prevent innominate artery erosion, respectively [5,6]. Although the generally preferred tracheal intervention site is between the second and third tracheal rings, there is no clear information on which spaces are safe. Since the site of intervention can only be determined by palpation or USG imaging without extensive surgical dissection in PDT, it has been reported that cannulation is usually performed from the second to fourth cartilage intervals with different PDT methods [7]. During the PDT procedure, according to the judgement and experience of the operator, the tracheal puncture site is determined by counting the tracheal rings after palpation of the thyroid prominence and cricoid cartilage from proximal to distal or tracking guidance of other anatomical markers. As the trachea moves caudally, it is displaced posteriorly and often to the right of the midline in the aortic arch, which could make the tracheal palpation at lower levels challenging [8]. It may be difficult to count the tracheal cartilages by palpation in patients with morbid obesity, short-thick neck, neck extension limitation, or in whom the trachea is not in the midline. Just as the length of the trachea varies between individuals, the length and structure of the cervical region where PDT can be performed are influenced by the morphologic characteristics of the patient, anatomic variations, structures having mass effect (thyroid gland, adipose tissue, etc.), and vascular structures in the region. Therefore, pre-procedural and procedural ultrasonography (USG) evaluation is usually preferred. Imaging-guided puncture is performed by clinicians who have an USG device and sufficient experience. However, difficulties in USG imaging in the group of patients with short neck, deep-settled trachea, or obesity may lead to misinterpretations of the tracheal intervals used in PDT procedures [7]. In such cases, the use of FOB may provide a more accurate determination of the tracheal intervals. Furthermore, the use of imaging modalities such as USG and FOB during PDT procedures is known to be effective in the prevention and early detection of tracheostomy-related complications, such as bleeding, loss of airway, false passage, pneumothorax, pneumomediastinum, and posterior wall injury [9,10]. However, USG or FOB may be unavailable due to a lack of equipment and/or operator experience. In this case, intervention can only be based on anatomical signs. A review of the literature showed that there are studies comparing the use of anatomical landmarks, USG, or FOB during PDT in terms of safety, duration, or development of complications [11,12,13]. However, no study was found that focuses on evaluating the effect of the morphological structure of individuals on the PDT intervention location and complications. The main objective of this study was to evaluate the percentage of agreement between the entry site observed by FOB and the prediction of the PDT level based on pre-procedural USG in PDT procedures performed using the forceps dilatation method.

## 2. Materials and Methods

### 2.1. Trial Design and Ethics

This is a prospective observational study that was conducted in the 33-bedded general ICUs of Akdeniz University Medical Faculty Hospital. The sample was collected from 30 April 2023 to 30 October 2023. The current study was carried out in accordance with the Declaration of Helsinki and approved by the Ethics Committee of Akdeniz University Faculty of Medicine, Antalya, Turkey (approval no: KAEK-274, dated 20 April 2022). In addition, this trial was registered in the Clinical Trials Registry at Clinicaltrials.gov (No. NCT05845775, dated 6 May 2023).

### 2.2. Participants

This study included patients who were 18 years of age or older, intubated, mechanically ventilated in the ICU, and scheduled for percutaneous dilatational tracheostomy (PDT). Patients who refused to participate in the study or their representative refused approval for PDT and who had a mass at the operation site for any reason, had skin infection at the procedure site, had coagulopathy, and had severe respiratory failure (Positive End Expiratory Pressure > 15 cm H_2_O, Fraction of Inspired Oxygen (FiO_2_) > 0.80) were excluded. Patient eligibility for PDT was reviewed, and informed consent was obtained from the patient and/or the patient’s relatives after detailed explanations about the study protocol.

### 2.3. Interventions

**Figure 1:**
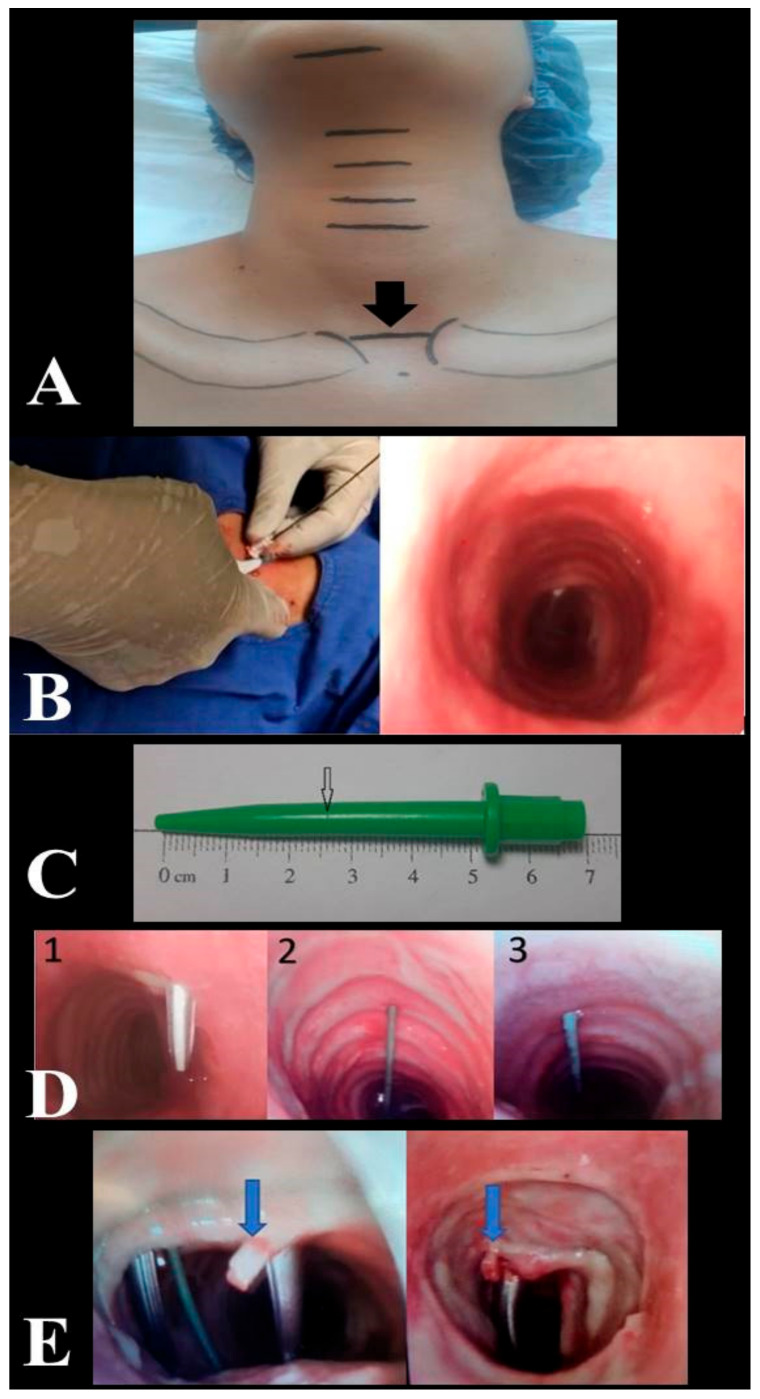
Anatomical landmark for site of intervention (**A**). Determination of the skin-to-trachea distance by fiberoptic bronchoscopy (**B**). Depth mark created with a scalpel in the dilator at skin level (**C**). Trachea puncture angle 1. Puncture angle > 30° --> repeat puncture. 2. Midline puncture. 3. Puncture angle < 30° (**D**). Cartilage fracture (**E**).

All PDT procedures were performed by intensivists with relevant experience (performed 50 or more procedures). All USG (Siemens, Munich, Germany) and fiberoptic bronchoscopy (FOB) (OlympusTM, Center Valley, PA, USA) procedures, evaluation of the puncture site, decision making to repeat tracheal puncture, recording of relevant data, and complications were performed by the same anaesthesiologist. Patients were administered vasoactive drugs to stabilize their hemodynamic conditions prior to and throughout the operation. In addition to standard haemodynamic monitoring, including five-lead electrocardiography and pulse oximetry monitoring, invasive arterial monitoring was performed throughout the operation. Patients received 2 mcg/kg fentanyl, 0.05 mg/kg midazolam, and 0.6 mg/kg rocuronium for induction of anaesthesia. Additional hypnotic and anaesthetic drugs were administered when required during the clinical follow-up. The mechanical ventilator was adjusted to provide 7 mL/kg tidal volume according to ideal body weight in pressure-controlled mode, and preoxygenation was achieved for 5 min with FiO_2_ = 1. While the patient’s neck was in neutral position, neck circumference was measured from the thyroid prominence and clavicle head levels. During the PDT procedure, the site of intervention was determined by the operator based on the imaginary line connecting the upper edge of the sternoclavicular joints as an anatomical landmark (Figure 1A). After the patient was given the standard PDT position, anatomical measurements (distances between the mentum and hyoid cartilage, hyoid cartilage and thyroid prominence, thyroid prominence and upper edge of the cricoid cartilage, lower edge of the cricoid cartilage and anatomical landmark, anatomical landmark and jugular notch and thickness of the cricoid cartilage) were performed with a soft length meter. The intervention site was evaluated by USG. The distance between the patient’s skin and the tracheal ring and the coronal diameter of the trachea on inspiration were measured by USG. The level of intercartilaginous puncture at the anatomical landmark was estimated by USG. To determine the skin-to-trachea distance, the dilator was marked at the skin level, while the tip of the dilator was seen in the trachea by FOB (Figure 1B). The jaws of forceps were manuplated in horizontal and vertical directions (Modified Griggs Forceps Dilatation method) in order to reduce the frequency of tracheal cartilage injury during dilatation of the puncture hole between the tracheal rings enough to accommodate the tracheostomy cannula [14,15]. The duration of the procedure, the puncture site, and number of interventions, the depth measurement obtained by marking the dilator used, and complications related to the procedure were recorded (Figure 1C). FOB was used to check the position of the needle in the trachea, the correct orientation of the guidewire, and to determine the site of puncture. If the degree of deviation in the tracheal puncture angle was >30°, the puncture was repeated targeting the midline, and the guidewire was introduced after confirming via FOB that the puncture was within the midline or less than 30° apart from the midline (Figure 1D).

### 2.4. Data Collection

Medical records of all included patients were prospectively analyzed. Demographic data (age, gender, body mass index), comorbidities, reason for ICU admission, APACHE-II score during hospitalization, anatomical measurements (distances between the mentum and hyoid cartilage, hyoid cartilage and thyroid prominence, thyroid prominence and upper edge of the cricoid cartilage, lower edge of the cricoid cartilage and anatomical landmark, anatomical landmark and jugular notch and thickness of the cricoid cartilage), length of ICU stay, and mechanical ventilation requirement were analyzed. The PDT intervention site estimated by pre-procedural USG and the PDT intervention site detected by FOB during the procedure were recorded for comparative analysis (evaluation of consistency). It was considered consistent when the PDT intervention site estimated by pre-pocedural USG and the intervention site observed by FOB during the procedure were at the same tracheal puncture level. Patients were followed-up for PDT complications, and the complications encountered were included in the analysis.

### 2.5. Outcomes

The primary outcome of this study was the percentage of consistency between the entry site observed with FOB and the prediction for the PDT level based on pre-procedural USG in PDT procedures performed using the forceps dilatation method. The secondary outcomes included the relationship between the PDT puncture site, intercartilaginous ligament level, and morphological measurements, as well as blood gas analysis of the patients before and after PDT and complications.

### 2.6. Statistical Analysis

#### 2.6.1. Sample Size

Prior research comparing ultrasound imaging and palpation for identifying the cricoid membrane indicated that a 20% increase in the success rate or a success rate of 70% or higher when utilizing ultrasound would be considered clinically significant for accurately locating the cricothyroid membrane [16]. Since there is no study comparing USG and FOB in this respect, we assumed that a consistency of 70% or greater between the two techniques would be clinically significant. Our pilot study revealed consistency between tracheostomy levels determined by USG and fiberoptic bronchoscope in 50% of patients. A minimum number of 65 patients were required to demonstrate a 20% improvement in consistency at a 90% power and 5% significance level. A total of 104 patients were recruited to the study over a period of six months to ensure sufficient power and to replace any dropouts.

#### 2.6.2. Data Analysis

All data collected in this study were recorded by the principal investigator, and the data were transferred to SPSS version 24 (SPSS Inc., Chicago, IL, USA) software for statistical analysis. Continuous variables were expressed as mean ± standard deviation or median values with minimum maximum and interquartile range (IQR); categorical variables were expressed as number and percentage. The compatibility of the data with normal distribution was evaluated by Kolmogorov–Smirnov test. In the case of normal distribution, independent and dependent quantitative data were analyzed with Student *t* test, and independent qualitative data were analyzed with chi-square test. When normal distribution was not achieved, Mann–Whitney U test was used for independent quantitative data, and Wilcoxon test was used for dependent quantitative data. Simple linear regression analysis was used to evaluate the relationships between PDT angulation levels determined by FOB and anatomical measurements. A Bland–Altman plot was created to show the difference between the levels determined by both methods against the means of the levels determined by FOB and USG. *p* < 0.05 values were considered statistically significant.

In this study, the minimum redundancy maximum relevancy (mRMR) algorithm was used to determine the parameters that can be related to cartilage fracture during the tracheostomy process. mRMR algorithm attempts to minimize redundancy between features while selecting the most relevant features related to the response [17]. The mRMR algorithm requires that a feature must satisfy two fundamental criteria in order to be considered a satisfactory representation of the response: minimal redundancy and maximal relevance. The algorithm measures redundancy and relevance using the mutual information of variables (pairwise mutual information of features and mutual information of a feature and the response). These features make the mRMR algorithm very useful in determining the features that best represent the response, especially in studies in medical research [18,19]. In response, the presence/absence of cartilage fractures was indicated, and 22 different features were presented to the mRMR algorithm. Features are arranged according to predictor importance scores. The analyses were carried out in MATLAB version R2019a (9.6.0.1072779).

## 3. Results

**Figure 2:**
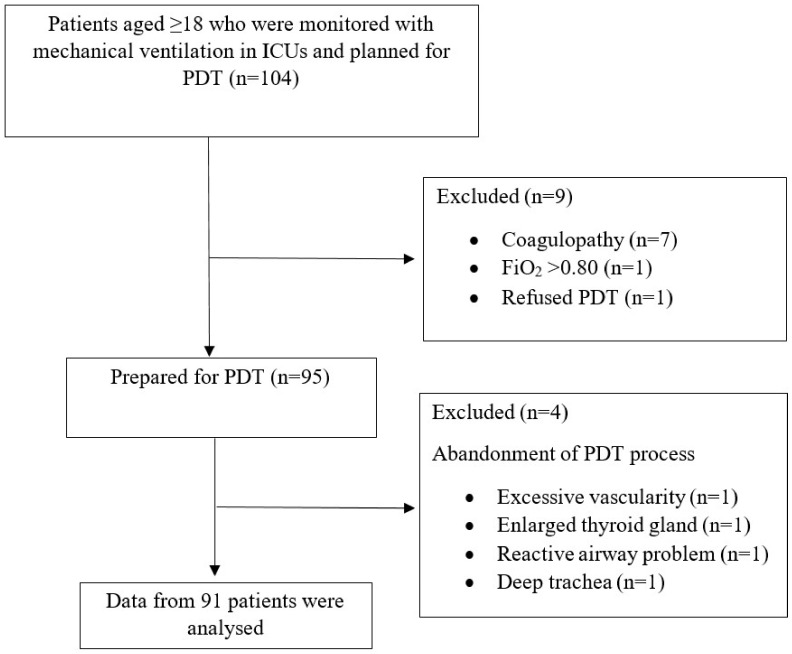
Flow diagram of the study.

A total of 104 patients were evaluated for this study. In nine patients who did not meet the inclusion criteria, the decision to perform PDT was canceled due to hypoxemic disorders, coagulopathy, or lack of consent. In four of the patients who were planned to be included in this study group and whose PDT preparations were completed, performing surgical tracheostomy rather than PDT was considered necessary after USG examination (thyroid mass, large vascular structure, overactive airway to stimuli, very deep trachea). As a result, data for 91 patients were collected and analyzed in accordance with the study protocol (Figure 2). All PDT procedures were performed by intensive care specialists. The demographic characteristics of the patients and ICU follow-up data are presented in Table 1.

**Figure 3:**
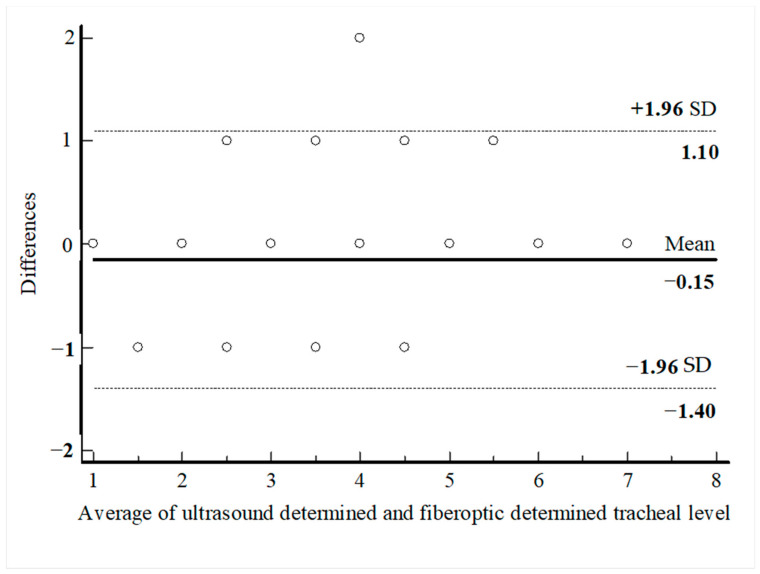
Bland–Altman analysis of the differences between ultasound-determined intercartilaginous puncture level and FOB-determined intercartilaginous puncture level.

**Figure 4:**
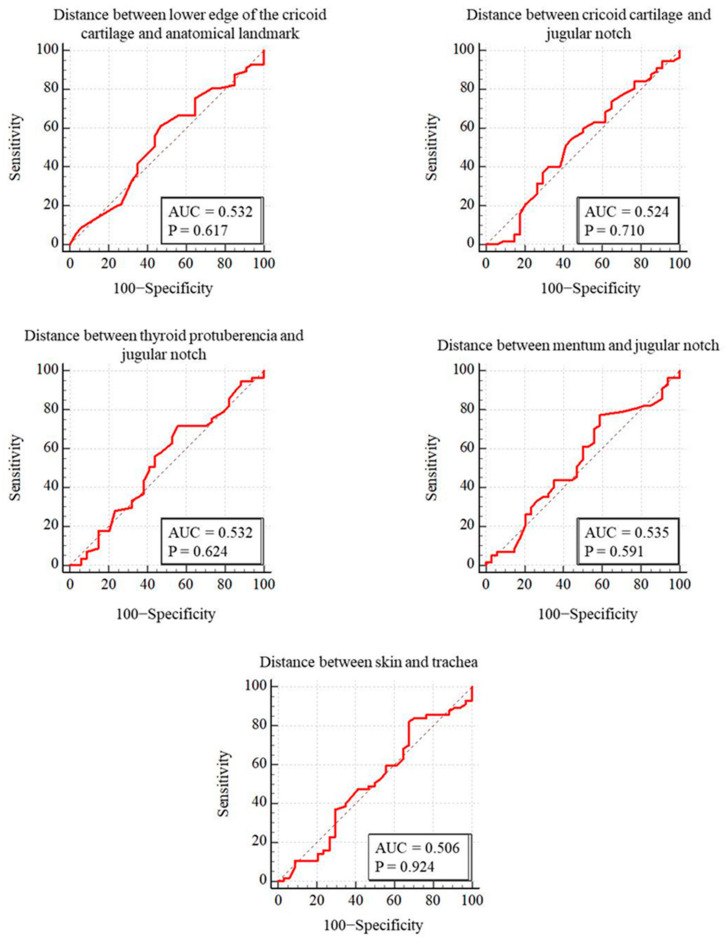
Comparison of ROC curves for puncture levels detected by FOB and ultrasound.

PDT was performed through the first intercartilaginous space in 9 patients, second in 37 patients, third in 30 patients, fourth in 9 patients, fifth in 3 patients, sixth in 2 patients, and seventh in 1 patient. In 57 patients (62.6%), the USG-estimated tracheal puncture level was consistent with the intercartilaginous space observed by FOB, which is considered as the gold-standard adjustment method, while in 34 patients (37.4%), there was a discrepancy between these two methods. Bland–Altman analysis was used to show the agreement between tracheal intervals determined by USG and FOB. In the Bland–Altman plot, the mean difference between tracheal intervals determined by USG and FOB was −0.15, and the 95% limits of agreement were found to be between −1.40 and 1.10 (Figure 3). According to this analysis, the tracheal puncture level determined by USG and FOB was in close agreement. There were no statistically significant differences in age, gender, body mass index, neck extension restriction, presence of tracheal deviation, and morphological measurements in patients with consistent or inconsistent USG and FOB (Table 2). ROC analyses were performed for morphological measurements according to the consistency of the levels recorded with FOB and USG. No morphologic measurement was a good predictor of the consistency of the tracheal puncture level determined by USG and FOB (Figure 4).

**Figure 5:**
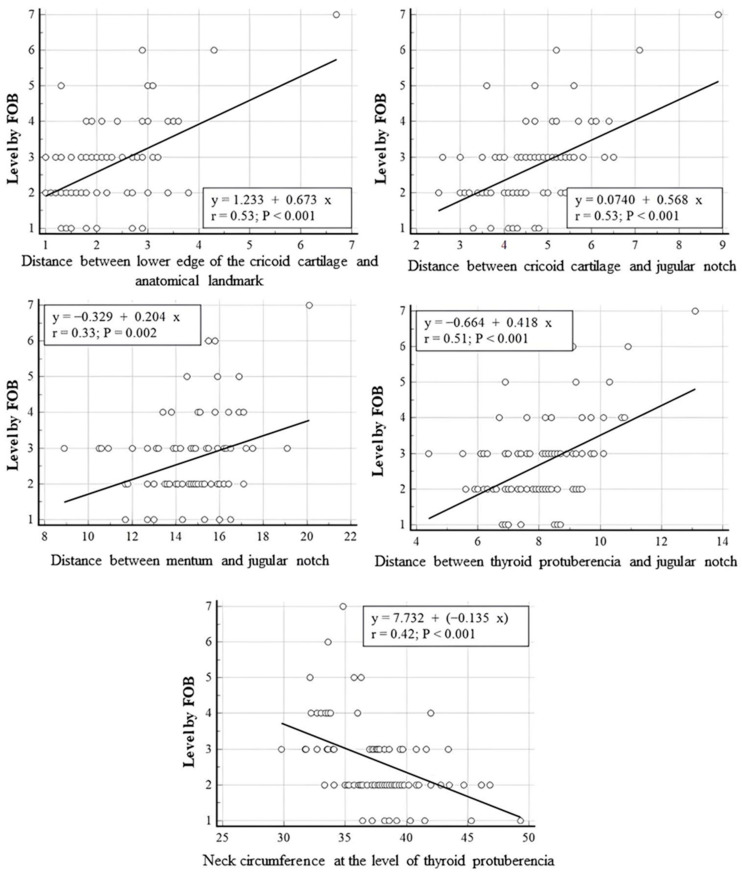
Linear regression analyses performed between PDT intercartilaginous puncture levels confirmed by FOB and anatomical measurements.

In linear regression analyses performed between PDT intercartilaginous puncture levels confirmed by FOB and anatomical measurements of the patients, a statistically significant positive linear relationship was found between the PDT-punctured intercartilaginous ligament level and the distances between cricoid cartilage and anatomical landmark, cricoid cartilage and jugular notch, mentum and jugular notch and thyroid protuberencia and jugular notch (r = 0.53, *p* = 0.001; r = 0.53, *p* = 0.001; r = 0.33, *p* = 0.002; r = 0.51, *p* = 0.001, respectively). A statistically significant negative linear correlation was found between the tracheal puncture level and neck circumference measured at the level of thyroid puberencia (r = 0.042, *p* < 0.001). The neck circumference measured from the level of thyroid prominence was inversely correlated with the intercartilageous ligament level at which PDT was introduced, while the distance between the mentum and jugular notch (neck length) and the distance between the cricoid cartilage lower edge and anatomic landmark were positively correlated with the PDT puncture level (*p* < 0.01). The correlation between PDT intercartilaginous puncture levels and anatomical measurements was moderate, except for the distance between the mentum and the jugular notch (weak correlation). Correlation strength was based on recent studies [20,21]. The scatter plots for linear regression analyses are presented in Figure 5. The regressing intercartilaginous puncture level against morphologic measurements using linear regression yielded a prediction of the intercartilaginous puncture level. The formulas for predicting the level of intercartilaginous puncture based on morphological measurements using linear regression are as follows.

Puncture level: 1.233 + (0.673 × Distance between lower edge of the cricoid cartilage and anatomical landmark)

Puncture level: 0.074 + (0.578 × Distance between cricoid cartilage and jugular notch)

Puncture level: −0.329 + (0.204 × Distance between mentum and jugular notch)

Puncture level: −0.664 + (0.418 × Distance between thyroid protuberencia and jugular notch)

Puncture level: 7.732 + (−0.135 × Neck circumference at the level of thyroid protuberencia)

Patients were further classified according to morphologic characteristics (obesity, short neck, thick neck, short thyromental distance) that may lead to difficult intubation and challenging PDT procedure, and the data obtained during the procedure were compared with the study population. The duration of the procedure was significantly higher in patients with a thick neck (*p* < 0.05). In patients with a short thyromental distance (<6 cm), the mean distance between the skin and trachea obtained by dilator marking and the mean procedure time were shorter than the overall mean (Table 3).

The tracheal puncture site determined by FOB was not significantly affected by the number of punctures or procedure duration (*p* > 0.05). In 63.7% of patients (58 patients), the first tracheal puncture was performed at or near the midline (<30°), and the guidewire was advanced into the trachea from this location. In 20 patients, PDT was performed with a single puncture, as the deviation from the midline was less than 30°. Of the remaining 33 patients, the second puncture was performed from the midline in 9 patients and near the midline in 12 patients. In ten patients (11%), tracheal puncture at or near the midline could be performed on the third attempt. There were two patients (2%) who required more than three attempts for appropriate tracheal puncture.

The results of arterial blood gas analysis of the patients before and after tracheostomy are presented in Table 4. While statistically significant adverse changes were observed in pH, partial pressure of carbondioxide (pCO_2_), and ratio of partial oxygen pressure to fraction of inspired oxygen (P/F) values between preoperative and postoperative measurements, no clinically significant ventilation or oxygenation deficiency developed.

**Figure 6:**
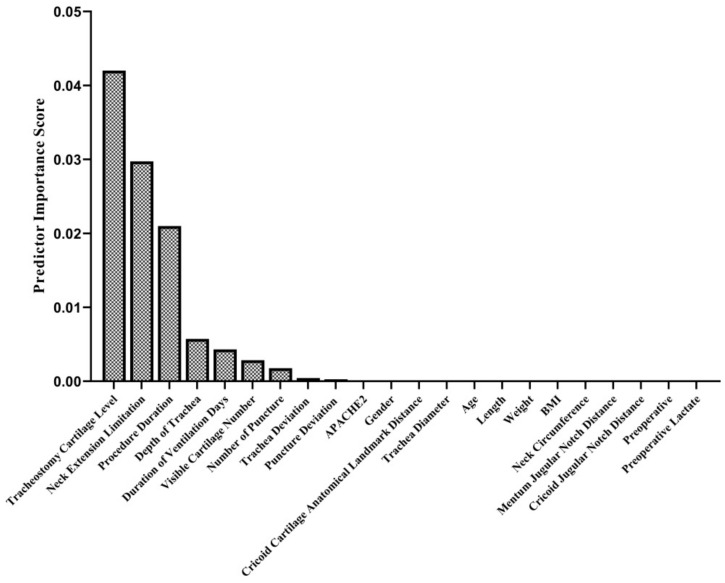
Predictor importance scores in terms of cartilage fracture during PDT. The relationship of 22 different variables was examined with the mRMR method to determine their relationship with cartilage fracture. This algorithm determines the relationship between inputs and responses based on mutual information. According to their mutual information scores, the algorithm ranks the input variables. The details of the algorithm are explained in the Methods section.

In the intraoperative period, no complications other than cartilage fracture (in 16 patients; 17.6%) (Figure 1E, Supplementary File), bleeding (11 patients; 12.1%), and desaturation (1 patient; 1.1%) were observed. In the postoperative period, bleeding occurred in 13 (14.3%) patients, subcutaneous emphysema developed in 1 (1.1%) patient, and infection around the tracheostomy was detected in 1 (1.1%) patient. The tracheostomy cartilage level, neck extension limitation, procedure duration, depth of trachea duration of ventilation days, visible cartilage number in USG imaging, and number of tracheal punctures were found to be predictors of cartilage fractures during the PDT procedure (Figure 6). In the late postoperative period, a tracheoesophageal fistula developed in one (1.1%) patient (Table 5).

## 4. Discussion

The results of our study revealed the most frequently used tracheal intervals in anatomical-measure-based PDT procedures, and the pre-procedural USG estimation of these tracheal intervals was consistent with the entry level confirmed by FOB in the majority of patients. In addition, we showed that there is a relationship between the morphological structure and cannula entry site.

A portion of the cervical trachea is located deep in the neck and can only be palpated to a limited extent. When the location of the trachea is tracked caudally, it displaces posteriorly, which may make tracheal palpation difficult at lower levels [8]. Therefore, it is usually not possible to determine the PDT intervention site by counting the tracheal intercartilaginous ligaments by palpation. It has been previously reported that USG-guided tracheal puncture during PDT procedures results in a lower number of needle interventions and a higher success rate of midline access compared with interventions based on anatomical landmarks [22,23]. However, a significant number of patients undergoing PDT may have difficulties in visualizing the actual needle and its trajectory during USG imaging due to obesity, large thyroid gland, and a short and thick neck structure [24,25,26]. In patients with tracheal deviation, it may also not be possible to perform simultaneous USG evaluation during a midline tracheal puncture due to difficulties in positioning the probe. Furthermore, USG has limited success in detecting complications such as posterior tracheal wall injuries and cartilage fractures due to its inability to visualise air interfaces [27,28]. The safest way to achieve tracheal access from the midline at the appropriate level of intercartilaginous ligaments is to approach the tracheal wall by dissecting the subcutaneous tissue under the incision with the help of blunt forceps and to ensure that the puncture is performed from the optimum location by performing FOB imaging simultaneously during the procedure [29,30]. In our study, tissue dissection was performed in all patients, the tracheal region to be punctured and the surrounding tissues (vascular structures, muscle and thyroid gland) were visualized, and the trachea was pulled to the midline when necessary to perform midline tracheal puncture. The aim of our study was not to investigate the reliability of USG-guided tracheal puncture or its effect on complications. For this reason, USG was not used to guide the puncture but to evaluate the morphologic structures before initiating the PDT procedure and to predict the location of the planned intervention between the tracheal rings in this study.

With the confirmation of the intervention site by FOB, it was observed that the level of intercartilaginous ligaments expected to be punctured in the pre-procedural USG examination according to the anatomical landmark and the actual punctured level of the trachea were not similar in 34 (37.4%) patients. Of the 18 patients with a deviated trachea, five tracheas were punctured one intercartilaginous ligament level down, three tracheas were punctured one intercartilaginous ligament level up, and one trachea was punctured two intercartilaginous ligament levels up. In patients without tracheal deviation, the rate of failure to puncture from the level estimated by USG was 30% (25 patients). Analysis of the available data showed that the difference in the level of tracheal puncture confirmed through FOB from that estimated by USG imaging was not related to tracheal deviation, midline retraction of the trachea, or the morphologic characteristics of the patient. Except for one patient, all punctures from different levels were performed from an upper or lower tracheal intercartilaginous ligament level. The low reliability of pre-procedural USG in predicting the tracheotomy insertion site may be due to the fact that the practitioner is able to palpate the tracheal rings with the help of dissection during the procedure and give the necessary angle to the needle tip, or because the planar course of the trachea may be misunderstood in 2D configuration with USG imaging. 

In our study, although the puncture was not performed under USG guidance, tracheotomy was performed in the second or third intercartilageous space in 67 (74%) patients, which are reported to be the most appropriate levels in the literature [29,31]. In nine patients with long necks, PDT was performed in the fifth to seventh tracheal interval, and in nine patients with short and thick necks, tracheotomy was performed in the first tracheal interval. There were no complications or procedural difficulties associated with the use of the upper or lower tracheal intercartilaginous ligaments in these patients. In a study conducted by Chung W. et al. tracheostomy was performed approximately 2 cm below the cricothyroid membrane using the Ciaglia Blue Rhino™ method [32]. They reported that PDT can be safely performed 2 cm below the cricothyroid membrane without the aid of a bronchoscope. In a subsequent report, they evaluated the feasibility of using the characteristics of patients who underwent three-dimensional computed tomography of the neck for different reasons to estimate the distance between the cricothyroid membrane and the second tracheal ring because the exact tracheostomy puncture site could not be determined in their previous study [33]. The results of their investigation showed that when performing PDT without bronchoscope guidance, the length of the cricoid cartilage to second tracheal ring depends on patient characteristics, especially height and gender. Similar to our study, Park et al. [33] examined the effect of patients’ morphologic characteristics on the tracheal level of PDT. However, the used anatomical landmark differed, and the patient’s gender and height were not found to be associated with the level of tracheal puncture, whereas the patient’s neck length and neck circumference measurements were shown to be associated with the level of tracheal puncture in our study. In the light of these findings, it is obvious that the puncture site for PDT may vary according to the morphologic characteristics of patients and should be evaluated before intervention. With the findings obtained in our study, it was possible to predetermine the tracheal level of the puncture to be performed in accordance with the anatomical landmark before PDT intervention by using different morphologic measurements, and this approach is reported for the first time in the literature.

The use of FOB during PDT has been shown to prevent complications, such as pneumothorax, subcutaneous emphysema, damage to the posterior tracheal wall, and to confirm appropriate endotracheal placement [34,35,36]. Our study also demonstrated that FOB imaging during the procedure allows for the repetition of tracheal puncture before dilatation of the tracheotomy tract when previous puncture was performed from inconvenient locations. Keeping the bronchoscope in the trachea during tracheal puncture can potentially damage the fiberoptic bronchoscope by the injector needle. However, in our clinical practice, the endotracheal tube (ETT) was pulled up to the first tracheal interval under imaging guidance with the FOB in its lumen, and the flexible shaft was kept in the ETT during the puncture. Injury caused by devices were prevented with this approach. All tracheal punctures were monitored in detail, and the needle entry level and proximity to the midline were recorded. Because of the possibility that the needle insertion site and cannulation deviation may increase the frequency of tracheal fracture, the puncture was repeated for deviations of more than 30° from the midline until providing midline puncture. Since the secondary aim of our study was to determine the optimum intercartilaginous levels for PDT in patients with different morphologic characteristics and the relationship between the levels and complications, repeated attempts were not performed to manage another puncture from the desired intercartilaginous space.

FOB may cause complications such as hypercarbia and hypoventilation during manipulation of the device’s working tube in the ETT [37,38]. In our study, while statistically significant adverse changes were observed in pH, pCO_2_, and P/F values between preoperative and postoperative measurements, no clinically significant ventilation or oxygenation deficiency developed. Blood gas changes may occur due to short-term ventilation reductions or interruptions during the procedure, blood pressure and perfusion disorders, and narrowing of the airway due to the presence of the working tube of the FOB. The actual effect of airway narrowing on blood gas values could not be estimated in our study due to the fact that all patients underwent FOB-guided PDT. However, the observed increase in PaCO_2_ values was similar to previously published data [39,40] and possibly due to the partial airway obstruction of the FOB with concomitant hypoventilation. Some authors recommend a risk–benefit assessment for each patient due to the risks of hypercarbia and subsequent respiratory acidosis and the use of larger ETT sizes (≥8 mm ID) during FOB used to mitigate this effect [41]. In our study, we performed FOB with an 8 mm ID ETT in our patients to avoid the possible adverse effects of hypercarbia and acidosis. The use of FOB during PDT procedures depends on the experience and preference of the clinic, considering the benefits of FOB in preventing complications that may develop during PDT and the risks, such as hypercarbia or hypoventilation.

Although bleeding remains one of the most common intraoperative complications during PDT, major bleeding is rare. Typically, the source of bleeding is the anterior jugular venous system, and small venous branches should be carefully checked as they can be a source of intraoperative and postoperative bleeding [42]. In our study, minor bleeding was detected in 11 patients during the PDT procedure and in 13 patients in the early postoperative period. Only one patient was treated with fresh frozen plasma in addition to compression. Bleeding was not associated with the morphologic characteristics of the patient, neck and trachea, PDT procedure (duration, number and location of punctures, etc.), and associated complications (cartilage fracture, desaturation). We believe that the identification of vascular structures with USG imaging before the procedure, application of adrenaline solution to the PDT tract before skin incision, and removal or tethering of vascular structures that have the potential to cause bleeding by dissection of subcutaneous tissues after incision were effective in preventing major bleeding complications. It has been reported that late PDT-related bleeding may be due to lateral or caudal placement of the cannula [7,8]. The reason for the absence of late postoperative major bleeding in our study may result from the fact that all tracheal dilatation and cannulations were performed close to the midline. Dilatation of the lateral wall of the trachea during the creation of a PDT tract results in the application of a downward force on the oblique wall of the anterolateral trachea. The change in the force vector applied to the tracheal wall may cause this force to proceed in a horizontal plane rather than perpendicular to the tracheal wall. This may result in paratracheal dilatation, resulting in damage to adjacent structures and airway loss or injury to the tracheal wall and cartilage fractures [43,44]. In our study, dilatation of the lateral wall of the trachea was not performed in any patient, and, thus, no complications of damage to surrounding tissues, airway loss, or pneumothorax were observed. Rudas et al. [7] reported that the simultaneous use of USG increased the success of midline puncture and, thus, the frequency of midline cannulation (87%) compared to patients without USG (50%). However, although the interventions during USG use were found to be more accurate than punctures using anatomical markers during their study, the researchers performed FOB imaging after guidewire insertion and then continued the procedure in the punctures found to be deviated and inserted the cannula from the lateral position (13%). In our study, even though the midline success during the first tracheal puncture was lower (64%) than the aforementioned study, the puncture was repeated with FOB imaging during the procedure, and dilatation and cannula placement were achieved from midline to near midline (<30°) (100%) in all patients. No adverse events (bleeding, tracheal damage, etc.) related to repeated punctures were encountered. Therefore, FOB visualization of the puncture was considered to be the most reliable way to prevent dilatation and cannulation of the trachea distant from the midline. 

The risk of tracheal cartilage fracture, a complication that can occur during PDT, increases at the upper levels of the trachea. Due to the backward angulation of the trachea at the thoracic inlet, additional force must be applied to widen the space between the cartilages at the upper level near the cricoid cartilage [45]. Since the cricoid cartilage is denser than other cartilage types, the force is transmitted to the lower cartilage, and the risk of cartilage fracture increases [46]. PDT at lower levels reduces the angle between the dilator and the trachea, so less force is needed [45]. In our study, a cartilage fracture was seen in 16 patients, and only 1 of these patients had a cartilage fracture in the first tracheal interval. The fact that the remaining 15 cartilage fractures occurred in the second and third intercartilaginous segments did not support the existing knowledge or assumptions in the literature. Cartilage fracture is an essential complication because of its potential to cause tracheal stenosis in the long term [47,48,49], but its actual frequency is not clearly identified because most PDT procedures are not visualized with FOB during the procedure. In our study, all PDT procedures from tracheal puncture to cannula placement were monitored by FOB, and, unlike many previously published studies, the most common peroperative complication was cartilage fracture, not minor bleeding [29,50,51,52]. For this reason, detailed research was conducted on morphologic features and procedural practices that may be related to cartilage fracture. The tracheostomy cartilage level, neck extension limitation, and procedure duration were found to be the most significant predictors for cartilage fracture during PDT. Although there is no morbidity due to cartilage fracture in the short term, studies are needed to evaluate the clinical significance of cartilage fracture since long-term complications have not been followed.

In our study, the distance between the skin and trachea was measured using two different methods. The first one is the indirect measurement made by USG imaging before PDT procedure. The second method is the direct measurement performed by marking the dilator at the skin level during tracheal dilatation, when the tip of the dilator is at the level of the tracheal wall. It was found that there was an average difference of 1.4 cm between the two measurement methods for each measurement. The discrepancy results from the fact that the ETT was in the trachea lumen at the visualized level of the trachea when the imaging was assessed with USG. This result is clearly demonstrated for the first time in the literature, showing that swelling of the ETT cuff in intubated patients may cause a margin of error in measurements to estimate the depth and diameter of the trachea by USG.

Our study has limitations. First of all, due to the limited frequency and type of complications recorded, the relationship between the morphologic structure and our PDT protocol and rare complications (pneumothorax, stoma infection, tracheal injury, tracheoesephageal fistula, etc.) could not be analyzed. Only the risk factors of cartilage fracture and bleeding complications could be investigated. The second limitation is that the patients were predominantly aged 60 and above, with cerebral ischemic or hemorrhagic injury. As our study population was inadequate to cover young patients and diseases that cause respiratory failure other than coma, the results may not be applicable to all patient groups. The third limitation is the inability to determine the long-term complications and morphologic features associated with these complications. The fourth limitation is that the angle of tracheal puncture deviation could not be determined with a precise numerical value. Since the determination of the angle by imaging and vectorial analysis would lead to a prolonged PDT time, a deviation below or above 30° was left to the practitioner’s estimation, and the decision to repeat the puncture was based on this estimation. All bronchoscopic follow-ups and puncture repeat decisions were assessed by the same practitioner.

In conclusion, the estimation of the PDT intervention level based on pre-procedure USG was consistent with the entry site observed by FOB in PDT procedures using the forceps dilatation method. The intercartilaginous puncture level could be estimated based on morphological measurements. The level of tracheal puncture during tracheal cannulation varies directly with neck length and inversely with neck circumference. The incidence of tracheal cartilage fracture could be accurately determined by simultaneous FOB imaging and was found to be the most common early complication. Future studies on this complication may support the findings of our study.

## Figures and Tables

**Table 1:** Demographic data and clinical characteristics (data are presented as mean ± standard deviation or n (%)).

Parameters	n = 91
Age, years	57.3 ± 19.7
Gender, male	66 (72.5%)
BMI, kg/m^2^	26.8 ± 4.6
APACHE-II score	21.2 ± 6.6
Comorbidities	
Hypertension	40 (43.9%)
Diabetes mellitus	38 (41.7%)
Chronic lung disease	20 (21.9%)
Chronic heart disease	11 (12%)
Chronic kidney disease	11 (12%)
Cerebrovascular disease	8 (0.8%)
Need for mechanical ventilation on admission	
No	26 (28.6%)
Yes	65 (71.4%)
Reason of ICU admission	
Neuromuscular disease	4 (4.4%)
Multitrauma (excluding cerebral damage)	7 (7.7%)
Ischemic/hemorrhagic brain injury	55 (60.4%)
Pulmonary disease	14 (15.4%)
Others	11 (12.1%)
Duration of mechanical ventilation before PDT, day	11.2 ± 5.8
Lenth of stay in ICU (entire duration), day	40.8 ± 23.5

BMI: Body mass index, APACHE-II: Acute Physiology And Chronic Health Evaluation, ICU: Intensive care unit, PDT: Percutaneous dilatational tracheostomy.

**Table 2:** Comparing variables according to whether the tracheal puncture level is consistent or not.

Variables	USG-FOB Inconsistent (n = 34)	USG-FOB Consistent (n = 57)	*p* Value
Age, years	59.4 ± 18.2	56 ± 20.6	0.426
Gender, male	22 (61.1%)	44 (80%)	0.070
BMI, kg/m^2^	27.2 ± 4	26.6 ± 4.9	0.550
Neck extension limitation	2 (5.6%)	5 (9.1%)	0.542
Tracheal deviation	9 (25%)	9 (16.4%)	0.312
Neck circumference (thyroid prominence level), cm	36.9 ± 2.8	37.8 ± 4	0.284
Neck circumference (clavicle level), cm	37.6 ± 3	38.4 ± 3.8	0.302
Distance between mentum and hyoid, cm	4.5 ± 0.7	4.5 ± 0.9	0.769
Distance between hyoid-thyroid prominence, cm	2.1 ± 0.7	2.2 ± 0.6	0.562
Between the thyroid prominence and the upper edge of the thyroid cartilage, cm	2.1 ± 0.6	2.1 ± 0.5	0.822
Cricoid cartilage thickness, cm	1.3 ± 0.3	2.1 ± 0.7	0.620
Distance between lower edge of cricoid cartilage and anatomical landmark, cm	2.1 ± 0.7	2.1 ± 0.9	0.922
Distance between anatomical landmark and jugular notch, cm	2.4 ± 0.5	2.5 ± 0.5	0.868
Distance between mentum and jugular notch, cm	14.6 ± 1.9	14.5-2.3	0.804
Distance between lower edge of cricoid cartilage and jugular notch, cm	4.6 ± 1.1	4.5 ± 1.1	0.978
Distance between mentum and upper edge of cricoid cartilage, cm	8.7 ± 1.5	8.8 ± 1.3	0.711
Distance between thyroid prominence and jugular notch, cm	8 ± 1.5	7.9 ± 1.4	0.836
Distance from skin to trachea, cm	1.4 ± 0.4	1.4 ± 0.3	0.874
Transverse diameter of the trachea, cm	1.8 ± 0.3	1.8 ± 0.2	0.626

USG: Ultrasonography, FOB: Fiberoptic bronchoscopy, BMI: Body mass index.

**Table 3:** Morphological features and anatomical measurements according to difficult intubation criteria.

Variables	All Patients (n = 91)	BMI > 30 kg/m²	Short Neck *	Thick Neck **	TM Distance < 6 cm	HM Distance < 4 cm
Number of patients	91	19	7	10	24	22
BMI, kg/m^2^	26.8 ± 4.6	33.4 ± 3.8	27.7 ± 3.6	28.8 ± 2.9	29.3 ± 6.1	28.6 ± 5.9
Height, cm	169.1 ± 10	164 ± 11.6	157.7 ± 10	177 ± 7	164.5 ± 12.2	167.5 ± 117
Neck circumference (thyroid prominence level), cm	37.4 ± 3.6	38.7 ± 4.8	36.4 ± 3.7	44.6 ± 2.3	36.6 ± 3.6	37.2 ± 3.4
TM distance, cm	6.68 ± 1.1	6.4 ± 1.1	4.95 ± 0.5	7.22 ± 1	5.6 ± 0.5	5.4 ± 0.8
HM distance, cm	4.5 ± 0.9	4.5 ± 0.9	3.51 ± 0.5	5.01 ± 1	3.6 ± 0.6	4.9 ± 0.4
Neck length, cm	14.71 ± 2	14.12 ± 2.2	10.87 ± 1	14.61 ± 1.4	12.8 ± 1.7	13.1 ± 1.9
Tracheal depth (USG), cm	1.4 ± 0.3	1.5 ± 0.3	1.6 ± 0.4	1.65 ± 0.4	1.46 ± 0.4	1.43 ± 0.4
Tracheal depth(dilator), cm	2.8 ± 0.4	3.05 ± 1	3.14 ± 1	3.37 ± 1.5	2.69 ± 0.8	2.63 ± 0.7
Number of punctures	2	2	1	1	1	1
Punture site (level of intercartilaginous)	2	2	3	2	3	3
Procedure time (sec)	460.3 ± 163	475.5 ± 163.5	451.4 ± 160	530.7 ± 250	424.5 ± 144	393.2 ± 128.6
Tracheal deviation	18 (19.8%)	5 (26.3%)	0	2 (20%)	4 (16.7%)	2 (9.1%)

Data are presented as mean±standard deviation or n (%). BMI: Body mass index, TM: Thyromental, HM: Hyomental, USG: Ultrasonography, * Sternomental distance < 12 cm, ** Circumference of neck, measured at the level of the thyroid prominence ≥ 42 cm.

**Table 4:** Blood gas analysis.

	Preoperative	Postoperative	*p* Value
pH *	7.51 ± 0.54	7.49 ± 0.07	0.006
PO_2_ **	125.5 ± 43.2	114.2 ± 37.4	0.009
PCO_2_ ***	33.3 ± 6.6	35.6 ± 9.3	0.008
Lactate	1.6 ± 0.9	1.6 ± 0.8	0.486
P/F ****	318.7 ± 132	279.4 ± 136.1	0.002

* pH: Potential Hydrogen, ** PO_2_: Partial O_2_ Pressure, *** PCO_2_: Partial CO_2_ Pressure, **** P/F: Partial O_2_ Pressure/FiO_2._

**Table 5:** Complications.

**Intraoperative Complications**	
Cartilage fracture	16 (17.6%)
Bleeding	11 (12.1%)
Desaturation	1 (1.1%)
**Postoperative Complications**	
Bleeding	13 (14.3%)
Subcutaneous emphysema	1 (1.1%)
Tracheostomy site infection	1 (1.1%)
Tracheoesophageal fistula	1 (1.1%)

## Data Availability

All data generated or analyzed during this study are included in this article. Further inquiries can be directed to the corresponding author.

## Supplementary Materials

The following supporting information can be downloaded at: https://www.mdpi.com/article/10.3390/jcm13102788/s1, Video S1: Cartilage fracture during percutaneous dilatational tracheostomy.

## References

[1] Khaja M., Haider A., Alapati A., Qureshi Z.A., Yapor L. (2022). Percutaneous Tracheostomy: A Bedside Procedure. Cureus.

[2] Ciaglia P., Firsching R., Syniec C. (1985). Elective percutaneous dilatational tracheostomy. A new simple bedside procedure; preliminary report. Chest.

[3] Sanabria A. (2014). Which percutaneous tracheostomy method is better? A systematic review. Respir. Care.

[4] Zouk A.N., Batra H. (2021). Managing complications of percutaneous tracheostomy and gastrostomy. J. Thorac. Dis..

[5] Sarper A., Ayten A., Eser I., Ozbudak O., Demircan A. (2005). Tracheal stenosis aftertracheostomy or intubation: Review with special regard to cause and management. Tex. Heart Inst. J..

[6] Epstein S.K. (2005). Late complications of tracheostomy. Respir. Care.

[7] Rudas M. (2012). The role of ultrasound in percutaneous dilatational tracheostomy. Australas. J. Ultrasound Med..

[8] Mehta C., Mehta Y. (2017). Percutaneous tracheostomy. Ann. Card. Anaesth..

[9] Saritas A., Kurnaz M.M. (2019). Comparison of Bronchoscopy-Guided and Real-Time Ultrasound-Guided Percutaneous Dilatational Tracheostomy: Safety, Complications, and Effectiveness in Critically Ill Patients. J. Intensive Care Med..

[10] Kost K.M. (2005). Endoscopic percutaneous dilatational tracheotomy: A prospective evaluation of 500 consecutive cases. Laryngoscope.

[11] Kang H.T., Kim S.Y., Lee M.K., Lee S.W., Baek A., Park K.N. (2022). Comparison Between Real-Time Ultrasound-Guided Percutaneous Tracheostomy and Surgical Tracheostomy in Critically Ill Patients. Crit. Care Res. Pract..

[12] Bermede O., Saricaoglu M.C., Baytas V., Hasde A.I., Inan M.B., Akar A.R. (2021). Percutaneous ultrasound-guided versus bronchoscopy-guided dilatational tracheostomy after median sternotomy: A case-control study. Turk. J. Thorac. Cardiovasc. Surg..

[13] Menegozzo C.A.M., Sorbello C.C.J., Santos J.P., Rasslan R., Damous S.H.B., Utiyama E.M. (2022). Safe ultrasound-guided percutaneous tracheostomy in eight steps and necessary precautions in COVID-19 patients. Rev. Colégio Bras. Cir..

[14] Elo G., Zubek L., Hargitai Z., Ivanyi Z., Branovics J., Gal J. (2012). Prevention of tracheal cartilage injury with modified Griggs technique during percutaneous tracheostomy—Randomized controlled cadaver study. Interv. Med. Appl. Sci..

[15] Bodis F., Orosz G., Toth J.T., Szabo M., Elo L.G., Gal J., Elo G. (2023). Percutaneous tracheostomy: Comparison of three different methods with respect to tracheal cartilage injury in cadavers-Randomized controlled study. Pathol. Oncol. Res..

[16] Siddiqui N., Yu E., Boulis S., You-Ten K.E. (2018). Ultrasound Is Superior to Palpation in Identifying the Cricothyroid Membrane in Subjects with Poorly Defined Neck Landmarks: A Randomized Clinical Trial. Anesthesiology.

[17] Ding C., Peng H. (2005). Minimum redundancy feature selection from microarray gene expression data. J. Bioinform. Comput. Biol..

[18] Remeseiro B., Bolon-Canedo V. (2019). A review of feature selection methods in medical applications. Comput. Biol. Med..

[19] Liu W., Cheng M., Li J., Zhang P., Fan H., Hu Q., Han M., Su L., He H., Tong Y. (2020). Classification of the Gut Microbiota of Patients in Intensive Care Units During Development of Sepsis and Septic Shock. Genom. Proteom. Bioinform..

[20] Akoglu H. (2018). User′s guide to correlation coefficients. Turk. J. Emerg. Med..

[21] Papageorgiou S.N. (2022). On correlation coefficients and their interpretation. J. Orthod..

[22] Kumar P., Kumar S., Hussain M., Singh R., Ahmed W., Anand R. (2022). Comparison of percutaneous tracheostomy methods in ICU patients: Conventional anatomical landmark method versus ultrasonography method—A randomised controlled trial. Indian J. Anaesth..

[23] Dugg K., Kathuria S., Gupta S., Gautam P.L., Singh T., Bansal H. (2022). Comparison of landmark guided and ultrasound guided percutaneous dilatational tracheostomy: Efficiency, efficacy and accuracy in critically ill patients. J. Anaesthesiol. Clin. Pharmacol..

[24] Alansari M., Alotair H., Al Aseri Z., Elhoseny M.A. (2015). Use of ultrasound guidance to improve the safety of percutaneous dilatational tracheostomy: A literature review. Crit. Care.

[25] Guinot P.G., Zogheib E., Petiot S., Marienne J.P., Guerin A.M., Monet P., Zaatar R., Dupont H. (2012). Ultrasound-guided percutaneous tracheostomy in critically ill obese patients. Crit. Care.

[26] Rajajee V., Fletcher J.J., Rochlen L.R., Jacobs T.L. (2011). Real-time ultrasound-guided percutaneous dilatational tracheostomy: A feasibility study. Crit. Care.

[27] Kundra P., Mishra S.K., Ramesh A. (2011). Ultrasound of the airway. Indian J. Anaesth..

[28] Tremblay L.N., Scales D.C. (2011). Ultrasound-guided tracheostomy—Not for the many, but perhaps the few… or the one. Crit. Care.

[29] Hashimoto D.A., Axtell A.L., Auchincloss H.G. (2020). Percutaneous Tracheostomy. N. Engl. J. Med..

[30] Gadkaree S.K., Schwartz D., Gerold K., Kim Y. (2016). Use of Bronchoscopy in Percutaneous Dilational Tracheostomy. JAMA Otolaryngol. Head Neck Surg..

[31] Ghattas C., Alsunaid S., Pickering E.M., Holden V.K. (2021). State of the art: Percutaneous tracheostomy in the intensive care unit. J. Thorac. Dis..

[32] Chung W., Kim B.M., Park S.I. (2016). Simply modified percutaneous tracheostomy using the Cook(R) Ciaglia Blue Rhino: A case series. Korean J. Anesth..

[33] Park J., Chung W., Song S., Kim Y.H., Lim C.S., Ko Y., Yun S., Park H., Park S., Hong B. (2019). Identifying the ideal tracheostomy site based on patient characteristics during percutaneous dilatational tracheostomy without bronchoscopy. Korean J. Anesth..

[34] Gobatto A.L.N., Besen B., Tierno P., Mendes P.V., Cadamuro F., Joelsons D., Melro L., Carmona M.J.C., Santori G., Pelosi P. (2016). Ultrasound-guided percutaneous dilational tracheostomy versus bronchoscopy-guided percutaneous dilational tracheostomy in critically ill patients (TRACHUS): A randomized noninferiority controlled trial. Intensive Care Med..

[35] Anon J.M., Arellano M.S., Perez-Marquez M., Diaz-Alvarino C., Marquez-Alonso J.A., Rodriguez-Pelaez J., Nanwani-Nanwani K., Martin-Pellicer A., Civantos B., Lopez-Fernandez A. (2021). The role of routine FIBERoptic bronchoscopy monitoring during percutaneous dilatational TRACHeostomy (FIBERTRACH): A study protocol for a randomized, controlled clinical trial. Trials.

[36] Shen G., Yin H., Cao Y., Zhang M., Wu J., Jiang X., Yu T., Lu W. (2019). Percutaneous dilatational tracheostomy versus fibre optic bronchoscopy-guided percutaneous dilatational tracheostomy in critically ill patients: A randomised controlled trial. Ir. J. Med. Sci..

[37] Yaghoubi S., Massoudi N., Fathi M., Nooraei N., Khezri M.B., Abdollahi S. (2020). Performing Percutaneous Dilational Tracheostomy without using Fiberoptic Bronchoscope. Tanaffos.

[38] Reilly P.M., Sing R.F., Giberson F.A., Anderson H.L., Rotondo M.F., Tinkoff G.H., Schwab C.W. (1997). Hypercarbia during tracheostomy: A comparison of percutaneous endoscopic, percutaneous Doppler, and standard surgical tracheostomy. Intensive Care Med..

[39] Grensemann J., Eichler L., Kahler S., Jarczak D., Simon M., Pinnschmidt H.O., Kluge S. (2017). Bronchoscopy versus an endotracheal tube mounted camera for the peri-interventional visualization of percutaneous dilatational tracheostomy—A prospective, randomized trial (VivaPDT). Crit. Care.

[40] Tariparast P.A., Brockmann A., Hartwig R., Kluge S., Grensemann J. (2022). Percutaneous dilatational tracheostomy with single use bronchoscopes versus reusable bronchoscopes—A prospective randomized trial (TraSUB). BMC Anesth..

[41] Karagiannidis C., Merten M.L., Heunks L., Strassmann S.E., Schafer S., Magnet F., Windisch W. (2019). Respiratory acidosis during bronchoscopy-guided percutaneous dilatational tracheostomy: Impact of ventilator settings and endotracheal tube size. BMC Anesth..

[42] Cipriano A., Mao M.L., Hon H.H., Vazquez D., Stawicki S.P., Sharpe R.P., Evans D.C. (2015). An overview of complications associated with open and percutaneous tracheostomy procedures. Int. J. Crit. Illn. Inj. Sci..

[43] Schmidt U., Hess D., Kwo J., Lagambina S., Gettings E., Khandwala F., Bigatello L.M., Stelfox H.T. (2008). Tracheostomy tube malposition in patients admitted to a respiratory acute care unit following prolonged ventilation. Chest.

[44] Polderman K.H., Spijkstra J.J., de Bree R., Christiaans H.M., Gelissen H.P., Wester J.P., Girbes A.R. (2003). Percutaneous dilatational tracheostomy in the ICU: Optimal organization, low complication rates, and description of a new complication. Chest.

[45] Karvandian K., Mahmoodpoor A., Mohammadi M., Beigmohammadi M., Jafarzadeh A. (2009). Tracheal cartilage fracture with the percutaneous dilatational tracheostomy, Ciaglia method. Middle East J. Anaesthesiol..

[46] Pauken C.M., Heyes R., Lott D.G. (2019). Mechanical, Cellular, and Proteomic Properties of Laryngotracheal Cartilage. Cartilage.

[47] Zias N., Chroneou A., Tabba M.K., Gonzalez A.V., Gray A.W., Lamb C.R., Riker D.R., Beamis J.F. (2008). Post tracheostomy and post intubation tracheal stenosis: Report of 31 cases and review of the literature. BMC Pulm. Med..

[48] Jacobs J.V., Hill D.A., Petersen S.R., Bremner R.M., Sue R.D., Smith M.A. (2013). “Corkscrew stenosis”: Defining and preventing a complication of percutaneous dilatational tracheostomy. J. Thorac. Cardiovasc. Surg..

[49] Jung Kwon O., Young Suh G., Pyo Chung M., Kim J., Han J., Kim H. (2003). Tracheal stenosis depends on the extent of cartilaginous injury in experimental canine model. Exp. Lung Res..

[50] Simon M., Metschke M., Braune S.A., Puschel K., Kluge S. (2013). Death after percutaneous dilatational tracheostomy: A systematic review and analysis of risk factors. Crit. Care.

[51] Shin D., Ma A., Chan Y. (2023). A Retrospective Review of 589 Percutaneous Tracheostomies in a Canadian Community Teaching Hospital. Ear Nose Throat J..

[52] Karasu D., Yılmaz C., Baytar Ç., Korfalı G. (2018). Retrospective Analysis of Percutaneous Tracheostomi Cases in Intensive Care Unit. Turk. J. Intensive Care.

